# Supplementary material for: Modeling glioblastoma heterogeneity as a dynamic network of cell states
Source: Mol Syst Biol. 2021 Sep 16;17(9):e10105. doi: 10.15252/msb.202010105 (PMC8444284; doi:10.15252/msb.202010105)
Supplement: Supplementary file 6 — Source Data for Figure 5 [file MSB-17-e10105-s004.zip › Figure5A_sourcedata/GSEA_3071/hallmarks_stateVI.GseaPreranked.1623742557031/HALLMARK_REACTIVE_OXYGEN_SPECIES_PATHWAY.html]

Details for gene set HALLMARK\_REACTIVE\_OXYGEN\_SPECIES\_PATHWAY[GSEA]

|  || Dataset | state63071 |
| Phenotype | NoPhenotypeAvailable |
| Upregulated in class | na\_pos |
| GeneSet | HALLMARK\_REACTIVE\_OXYGEN\_SPECIES\_PATHWAY |
| Enrichment Score (ES) | 0.40079898 |
| Normalized Enrichment Score (NES) | 1.6271372 |
| Nominal p-value | 0.02846975 |
| FDR q-value | 0.042136643 |
| FWER p-Value | 0.188 |
Table: GSEA Results Summary

  

Fig 1: Enrichment plot: HALLMARK\_REACTIVE\_OXYGEN\_SPECIES\_PATHWAY      
 Profile of the Running ES Score & Positions of GeneSet Members on the Rank Ordered List

  

| PROBE | GENE SYMBOL | GENE\_TITLE | RANK IN GENE LIST | RANK METRIC SCORE | RUNNING ES | CORE ENRICHMENT || 1 | TXN |  |  | 209 | 0.597 | 0.0233 | Yes |
| 2 | MGST1 |  |  | 298 | 0.543 | 0.0727 | Yes |
| 3 | GLRX |  |  | 330 | 0.523 | 0.1351 | Yes |
| 4 | GLRX2 |  |  | 382 | 0.496 | 0.1883 | Yes |
| 5 | PFKP |  |  | 473 | 0.463 | 0.2263 | Yes |
| 6 | GPX3 |  |  | 493 | 0.457 | 0.2830 | Yes |
| 7 | PDLIM1 |  |  | 524 | 0.449 | 0.3357 | Yes |
| 8 | PRDX4 |  |  | 647 | 0.412 | 0.3579 | Yes |
| 9 | TXNRD2 |  |  | 894 | 0.361 | 0.3390 | Yes |
| 10 | ERCC2 |  |  | 949 | 0.353 | 0.3720 | Yes |
| 11 | LSP1 |  |  | 1048 | 0.337 | 0.3908 | Yes |
| 12 | G6PD |  |  | 1170 | 0.320 | 0.4008 | Yes |
| 13 | PRNP |  |  | 1866 | -0.260 | 0.2442 | No |
| 14 | CDKN2D |  |  | 2548 | -0.387 | 0.1088 | No |
| 15 | GPX4 |  |  | 2913 | -0.499 | 0.0761 | No |
| 16 | PRDX2 |  |  | 3409 | -0.912 | 0.0632 | No |
Table: GSEA details [plain text format]

  

Fig 2: HALLMARK\_REACTIVE\_OXYGEN\_SPECIES\_PATHWAY: Random ES distribution      
 Gene set null distribution of ES for **HALLMARK\_REACTIVE\_OXYGEN\_SPECIES\_PATHWAY**
